# Thalamic morphology predicts the onset of freezing of gait in Parkinson’s disease

**DOI:** 10.1038/s41531-021-00163-0

**Published:** 2021-03-02

**Authors:** Nicholas D’Cruz, Griet Vervoort, Sima Chalavi, Bauke W. Dijkstra, Moran Gilat, Alice Nieuwboer

**Affiliations:** 1 KU Leuven, Department of Rehabilitation Sciences, Neurorehabilitation Research Group, B-3000 Leuven, Belgium; 2 KU Leuven, Department of Movement Sciences, Movement Control & Neuroplasticity Research Group, B-3000 Leuven, Belgium

**Keywords:** Basal ganglia, Predictive markers, Parkinson's disease

## Abstract

The onset of freezing of gait (FOG) in Parkinson’s disease (PD) is a critical milestone, marked by a higher risk of falls and reduced quality of life. FOG is associated with alterations in subcortical neural circuits, yet no study has assessed whether subcortical morphology can predict the onset of clinical FOG. In this prospective multimodal neuroimaging cohort study, we performed vertex-based analysis of grey matter morphology in fifty-seven individuals with PD at study entry and two years later. We also explored the behavioral correlates and resting-state functional connectivity related to these local volume differences. At study entry, we found that freezers (*N* = 12) and persons who developed FOG during the course of the study (converters) (*N* = 9) showed local inflations in bilateral thalamus in contrast to persons who did not (non-converters) (*N* = 36). Longitudinally, converters (*N* = 7) also showed local inflation in the left thalamus, as compared to non-converters (*N* = 36). A model including sex, daily levodopa equivalent dose, and local thalamic inflation predicted conversion with good accuracy (AUC: 0.87, sensitivity: 88.9%, specificity: 77.8%). Exploratory analyses showed that local thalamic inflations were associated with larger medial thalamic sub-nuclei volumes and better cognitive performance. Resting-state analyses further revealed that converters had stronger thalamo-cortical coupling with limbic and cognitive regions pre-conversion, with a marked reduction in coupling over the two years. Finally, validation using the PPMI cohort suggested FOG-specific non-linear evolution of thalamic local volume. These findings provide markers of, and deeper insights into conversion to FOG, which may foster earlier intervention and better mobility for persons with PD.

## Introduction

Freezing of gait (FOG) is a debilitating symptom affecting many people with Parkinson’s disease (PD), defined as a ‘brief, episodic absence or marked reduction of forward progression of the feet despite the intention to walk’^1^. This leads to an increased risk of falling^2^, and a major impact on physical and mental health-related quality of life^3,4^. Notoriously, FOG is challenging to treat due to the differential response to dopamine-replacement therapy and the potential negative effects of levodopa on FOG with time^5,6^. In addition, the effects of rehabilitation are short-term^7^ and only possible when FOG is still mild^8–10^. Given these challenges, markers of FOG onset are essential to screen for FOG conversion and deliver therapy pre-emptively.

Until now, various studies have looked into the clinical risk factors of conversion to FOG^11–17^. However, only one study investigated neural markers of conversion, and found that striatal dopaminergic deficits additionally contributed to a clinical prediction model of this transition^18^. Owing to the limited evidence, an investigation into neural markers of conversion is warranted for additional predictive purposes and to help explain the elusive mechanisms leading to the onset of FOG^19^. While freezing episodes likely signify transient dysfunctional information processing across the cortico-basal ganglia-thalamo-cortical parallel circuits^20–22^, these transient events are challenging to elicit and interpret, and therefore have poor applicability as predictive markers. Compensatory or maladaptive structural alterations of the nodes within these circuits, however, are more persistent and therefore easier to capture as possible predictive markers for FOG in PD. A recent systematic review^23^ of cross-sectional investigations of structural alterations in persons with PD and FOG revealed widespread cortical and subcortical grey matter atrophy^24–33^, and associations between FOG severity and atrophy^24,29,31^, suggesting that grey matter morphology may be a useful marker of FOG onset and progression. Furthermore, when centrally or peripherally originating PD pathology enters the brain^34^, subcortical structures are affected earlier^35,36^, and maybe early markers of FOG onset. However, a prospective assessment of this hypothesis is lacking.

**Fig. 1:**
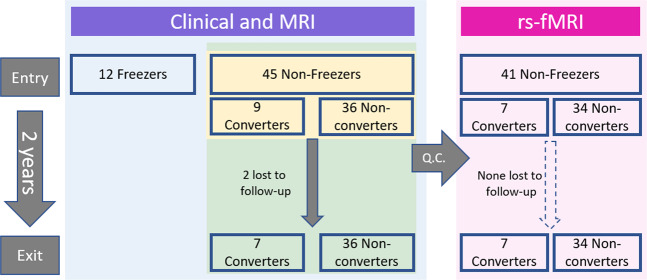
Study flowchart and analyses. Forty-five non-freezers were included and followed up over two years. Of these, 9 participants developed FOG during these two years and were classified as converters. Twelve freezers were also included as a positive control group at study entry. Colored boxes indicate participants included in the main analysis (blue), prediction analysis (yellow), correlation analyses with clinical measures and thalamic sub-nuclei volumes (green), and functional connectivity analyses (pink). *MRI* Magnetic Resonance Imaging, *rs-fMRI* resting state functional MRI, *Q.C.* quality control for high motion.

In this longitudinal study, we thus investigated the FOG-related morphological changes in key subcortical structures using magnetic resonance imaging (MRI) (Fig. 1). We compared local and global volume differences at study entry and over two years between participants that did not present with or develop FOG during the study (non-converters) and those that either presented with FOG at study entry (freezers) or developed FOG during the study (converters). Based on previous cross-sectional work^29,32,33^, we hypothesized that individuals with, or about to develop, FOG, would show local or global volume decline in the brainstem, thalamus, and/or caudate compared to non-converters and that this might serve as a neural marker of conversion. We then assessed how well structural markers at study entry could predict conversion to FOG over two years and explored their behavioral associations and underlying sub-nuclear volumetric and functional correlates. Finally, initial validation of these markers was performed using data from the Parkinson’s Progression Markers Initiative (PPMI) cohort.

## Results

### Participants

Out of the fifty-seven persons with PD enrolled in the study, forty-five had never experienced FOG at study entry (non-freezers), while twelve participants already presented with FOG (freezers). Over the two years, nine (20%) of the forty-five non-freezers converted to FOG (converters). All but two of the participants (both converters) completed the two clinical and imaging assessments. One converter dropped out of the study after the first follow-up due to co-morbidities, and the second converter performed all behavioral testing but did not undergo imaging at study exit.

### Demographics

Behavioral analysis at study entry revealed that the nine to-be converters had higher daily levodopa dose (*P* = 0.050), and higher (worse) MDS-UPDRS part I ‘non-motor activities of daily living’ (*P* = 0.042) and total scores (*P* = 0.015) than the to-be non-converters. Freezers, on the other hand, had longer disease duration (P = 0.036), worse scores on part II ‘motor activities of daily living’ (*P* < 0.001), part III ‘motor examination’ (*P* = 0.001), and total score (*P* < 0.001) of the MDS-UPDRS, higher likelihood to have fallen in the previous six months (*P* = 0.021), and lower (worse) balance scores on the MiniBEST (*P* = 0.004) when compared to to-be non-converters. Freezers also had worse balance scores on the MiniBEST (*P* = 0.005) and needed longer time to complete the alternate naming test (*P* = 0.045) compared to to-be converters (Table 1).

**Table 1:** Demographic differences between sub-groups at study entry.

	Non-converters	Converters	Freezers	P^1,2^	P^3^	P^3^	P^3^
					Non-converters – Converters	Non-converters – Freezers	Converters – Freezers
*N*	36	9	12				
Age	59 (14.3)	57 (17.5)	69 (11.5)	0.107			
Gender (F)	7 (47.2%)	4 (44 %)	1 (8.3%)	0.052			
Disease Duration (Years)	5 (5.1)	7 (5.5)	7.5 (3.8)	0.021		0.036	
LEDD	412.5 (313)	814 (452)	639 (311)	0.032	.050		
MDS-UPDRS Part I	9 (4)	15 (11)	9 (8.3)	0.046	.042		
MDS-UPDRS Part II	8.5 (6.8)	10 (12)	16.5 (14.8)	<0.001		<0.001	
MDS-UPDRS Part III	23.5 (14.3)	30 (10.5)	39 (12.5)	0.001		0.001	
MDS-UPDRS Part IV	1 (3.5)	2 (4.5)	0 (2)	0.291			
MDS-UPDRS Total	43.5 (21)	62 (22.5)	70.5 (33.3)	<0.001	.015	< .001	
Hoehn & Yahr (I / II / III)	9/21/6	0/7/1	0/9/2	0.062			
MOCA (0–30)	27 (3)	28 (3.5)	25.5 (4.8)	0.434			
FAB (0–18)	16 (1.8)	16 (2.5)	15 (3)	0.045			
Trail Making Test B-A (s)	38.3 (30)	41.7 (39.6)	52 (65)	0.302			
Alternate Naming Test (s)	21 (11.6)	14.1 (11.5)	26 (24.7)	0.033			0.045
Figure of Rey Copy (s)	102 (48)	80 (38.7)	115.5 (96.2)	0.256			
Figure of Rey Recall	21 (13.5)	23.5 (7.5)	22 (14)	0.438			
HADS Anxiety (0–21)	6 (7.5)	6 (9.5)	6 (3.8)	0.889			
HADS Depression (0–21)	4.5 (4.8)	6 (4)	6 (6.5)	0.193			
Fallers	4 (11%)	4 (44%)	6 (50%)	0.009		0.021	
MiniBEST score (0–28)	25 (4)	26 (2)	22 (6.8)	0.002		0.004	0.005

Group median values (interquartile range) or number (percentage) along with *p*-values reported. 1: One-way ANOVA with Group as Main Effect for scale measures, 2: χ2 Likelihood Ratio for class measures, 3: Bonferroni corrected post-hoc pairwise tests. *LEDD* Daily Levodopa Equivalent Dose, *Non-LED* Non Levodopa medication usage, *MDS-UPDRS* Movement Disorder Society sponsored revision of the Unified Parkinson’s Disease Rating Scale, *MOCA* Montreal Cognitive Assessment, *FAB* Frontal Assessment Battery, *HADS* Hospital Anxiety and Depression Scale, *MiniBEST* Mini Balance Evaluation Systems Test. Note: All non-converters and converters were non-freezers at study entry. Sub-group assignments were based on FOG classification at study exit.

### Local volume differences at study entry and over two years

**Fig. 2:**
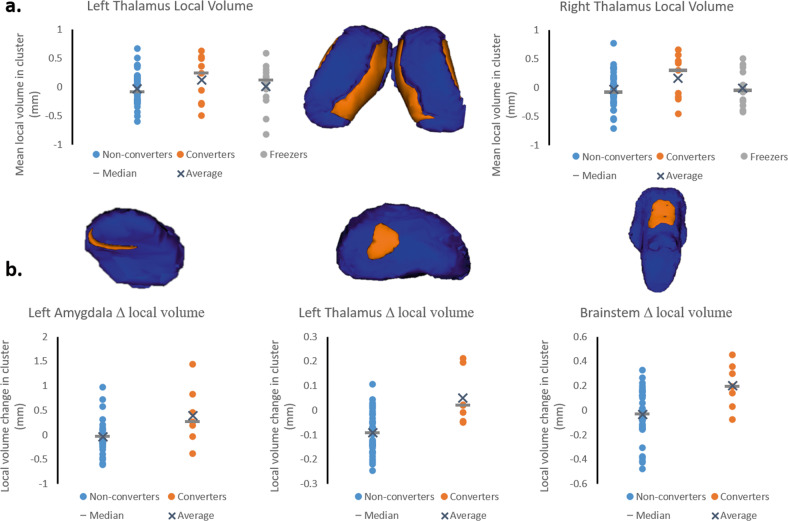
Local volume alterations at study entry and over two years. Surface models of subcortical structures (in blue) are depicted with significant clusters (in orange) showing local volume alterations at study entry (**a**) and over two years (**b**). Residual local volume for each participant within thresholded clusters and *p*-values for the group effect from the ANCOVA accounting for age, gender, and daily levodopa dose is plotted along with median and average group values. **a**. At study entry, significant differences in local volume were found in the thalamus bilaterally. Post-hoc tests revealed that converters and freezers showed local inflations in the thalamus clusters relative to non-converters. **b**. Over the two years, significant changes were observed in local volume in the brainstem, and trends towards significance in the left amygdala and left thalamus. Converters showed local volume inflation in the brainstem, left amygdala, and left thalamus, while non-converters showed deflation (see text for details).

At study entry (*N* = 57), freezers and converters showed significant local shape inflations compared to non-converters in the right (*P*_FWE min_ = 0.012) and left (*P*_FWE min_ = 0.006) thalamus. Voxels with *P*_FWE_ < 0.05 were thresholded to create the two clusters reported in Fig. 2a and Supplementary Table 1. The mean local volume value for each cluster was extracted to perform post-hoc testing. Post-hoc tests revealed that both converters and freezers showed local inflations in each cluster compared to non-converters with only slight variation in the extent across the two clusters (Fig. 2a).

Longitudinaly (*N* = 43), permutation testing revealed that converters and non-converters showed significantly different local shape alterations over two years in the brainstem (*P*_FWE max_ = 0.045), and clusters approaching significance in the left thalamus (*P*_FWE max_ = 0.06) and left amygdala (*P*_FWE max_ = 0.083). Voxels were thresholded at *P*_FWE_ < 0.1 to give the clusters reported in Fig. 2b and Supplementary Table 1. In each of these clusters, the direction of each of these local shape alterations was different between converters (inflation) and non-converters (deflation) (Fig. 2b).

### Global volume differences at study entry and over two years

At study entry (*N* = 57), differences in global volumes with a trend towards significance were found in the brainstem (*P* = 0.052). Post-hoc tests revealed that freezers had significantly smaller brainstem volumes compared to non-converters (*P* = 0.034), while no differences were found between converters and non-converters (*P* = 0.286). Longitudinaly (*N* = 43), non-converters showed a significantly greater decline in global volume over the two years in the right pallidum (*P* = 0.050), left thalamus (*P* = 0.036), and brainstem (*P* = 0.026); and a trend towards significance in the right thalamus (*P* = 0.084), compared to converters (Table 2).

**Table 2:** Differences in global volumes of the subcortical structures.

A. At study entry				
Structure	Non-converters	Converters	Freezers	*P*
Brainstem	25282 (323)	24980 (658)	23471 (583)	0.052

B. Over two years				
Structure	Non-converters	Converters		*P*
Left Thalamus	−128 (36)	88 (89)		0.036
Right Thalamus	−111 (31)	40 (76)		0.084
Right Pallidum	−40 (20)	70 (49)		0.050
Brainstem	−384 (94)	208 (230)		0.026

Estimated marginal means (standard error) from ANCOVA accounting for the effects of age, sex, daily levodopa dose, and total intracranial volume are reported in cubic mm for nuclei showing global volume differences – F-test *p*-values < 0.1. A. In the brainstem, post-hoc tests at study entry were significant between non-converters and freezers (*P* = 0.034), but not between non-converters and converters (*P* = 0.286). B. Over the two years, non-converters showed a decrease in global volume in the bilateral thalamus, the right pallidum, and the brainstem, compared to converters.

### Prediction of FOG conversion in non-freezers at study entry

**Fig. 3:**
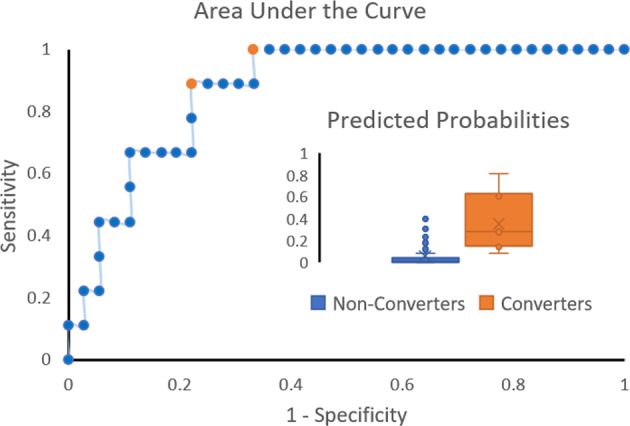
Prediction of conversion to FOG over two years. Area under the curve of the FOG conversion model including sex, daily levodopa equivalent dose, and left thalamus local volume is shown. The area under the curve of the model was 0.87 and the Brier score was 0.11. Optimum performance was achieved at two Youden’s points (in orange), probability cutoffs of 0.09 (sensitivity: 100%, specificity: 66.7%) and 0.19 (sensitivity: 88.9%, specificity: 77.8%). Box and whisker plots of predicted probabilities are also shown for non-converters and converters along with median (horizontal line) and mean (x), showing good separation of the two groups (inset).

Backward logistic regression retained gender (Odds Ratio for females = 0.074, 95%CI = 0.005–1.11, *P* = 0.06), daily levodopa equivalent dose (Odds Ratio = 1.006, 95%CI = 1.002–1.01, *P* = 0.006), and the cluster from the left thalamus (Odds Ratio = 383.45, 95%CI = 3.41–43124, *P* = 0.014) as significant predictors of conversion to FOG (*N* = 45). The model was able to predict conversion with an AUC of 0.87 (1 is ideal) and Brier score of 0.11 (0 is ideal), indicating good performance, with a sensitivity of 88.9% and specificity of 77.8% at a Youden’s point – a probability cutoff of 0.19 (Fig. 3). Bootstrap resampling the model selection and evaluation led to similar estimates (Supplementary Table 2).

### Local volume associations with behavior

In the group of converters and non-converters, significant and consistent associations with various cognitive tests were found at study entry (Supplementary Table 3) (max *N* = 45), whereby greater inflation in the thalamic clusters related to better cognitive performance (TMTB-A: Left/Right Thalamus – *r* = −0.34/−0.38, Alternate Naming Test time: Left/Right Thalamus – *r* = −0.43/−0.32, and Figure of Rey copy time: Left/Right Thalamus – *r* = −0.33/−0.40). Conversely, significant associations were found with worse scores on the MDS-UPDRS part IV (Left/Right Thalamus – *r* = 0.43/0.44), particularly items related to the amount of time spent in OFF (Left/Right Thalamus – *r* = 0.37/0.36), functional impact of ON/OFF fluctuations (Left/Right Thalamus – *r* = 0.38/0.39), and predictability of OFF episodes (Left/Right Thalamus – *r* = 0.5/0.53). No significant correlations were found between clinical change scores and change in local volumes (max *N* = 43).

### Association between local thalamic shape and thalamic sub-nuclei volume

**Fig. 4:**
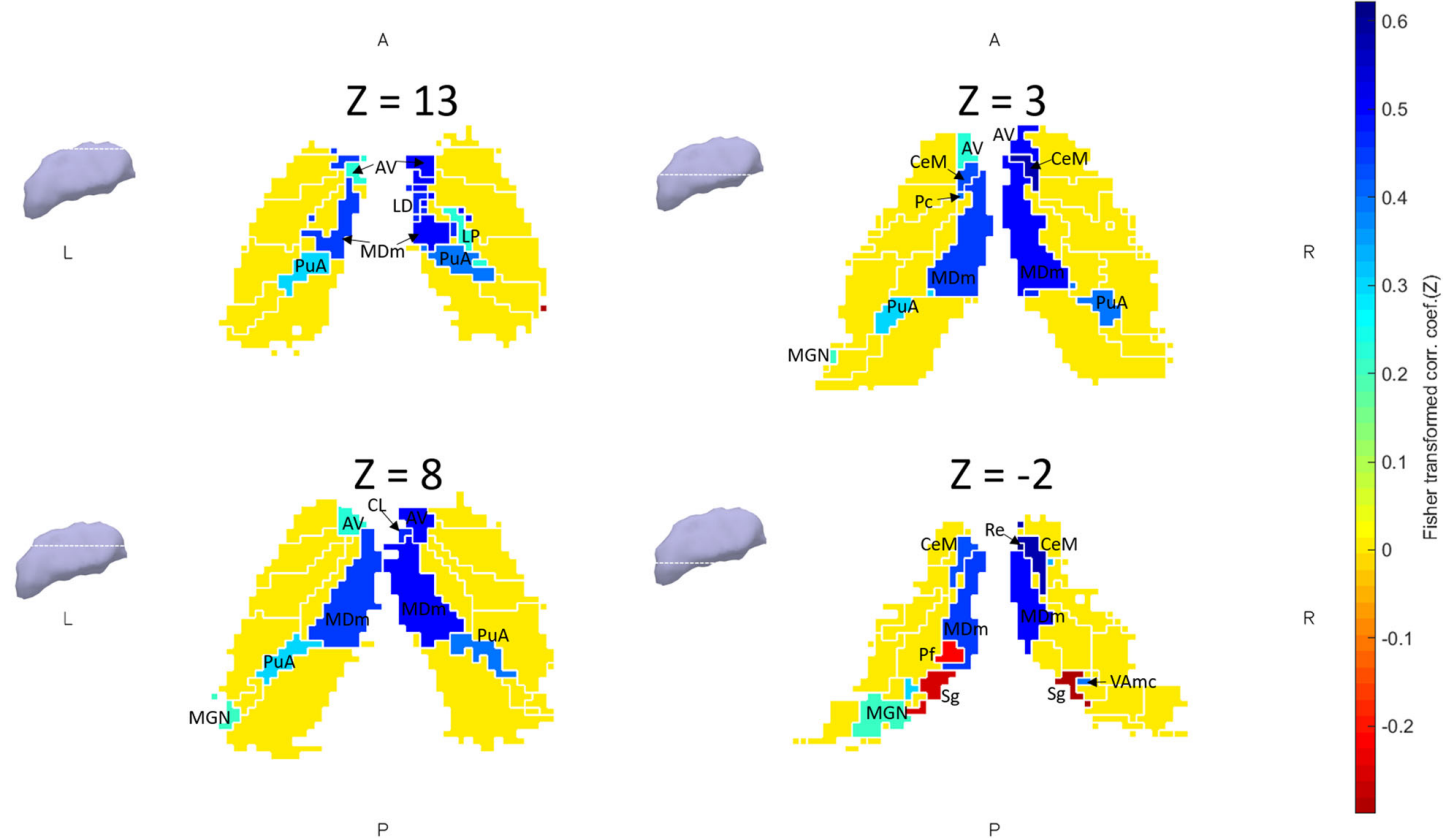
Thalamus sub-nuclei volumes related to local volume inflations. Axial slices (5 mm apart) through the thalamus showing the association between the sub-nuclei volumes and the extent of local deformation in the significant clusters at study entry. Pearson product-moment correlation was performed between sub-nuclei volume and local volume values in the two significant clusters. Pearson’s r-values > ± 0.2 for each side (left thalamus local volume with left thalamus sub-nuclei, and similarly for the right side) are depicted on a single subject thalamus segmentation in MNI space. Inferiorly located nuclei were inversely associated, while medial nuclei were positively associated with the extent of local deformation. *AV* anteroventral, *LD* laterodorsal, *LP* lateral posterior, *CeM* Central Medial, *CL* Central Lateral, *Pc* Paracentral, *Pf* parafascicular, *MDm* mediodorsal medial magnocellular, *MGN* medial geniculate, *Re* Reuniens, *Sg* suprageniculate, *PuA* pulvinar anterior, *VAmc* Ventral Anterior magnacellular.

At study entry (*N* = 45), we found significant positive associations between local shapes of the two thalamic clusters and volumes of a medial group of nuclei (bilaterally) comprising the anteroventral, central medial, lateral dorsal, reuniens, paracentral, mediodorsal (magnocellular) and the anterior pulvinar. An inverse association was revealed with the suprageniculate volume (Fig. 4). Changes in local volume over the two years (*N* = 43) were positively associated with the left paracentral volume change and negatively associated with the right suprageniculate volume change (Supplementary Table 4).

### rsfMRI functional connectivity between thalamic sub-nuclei and cortical areas

**Fig. 5:**
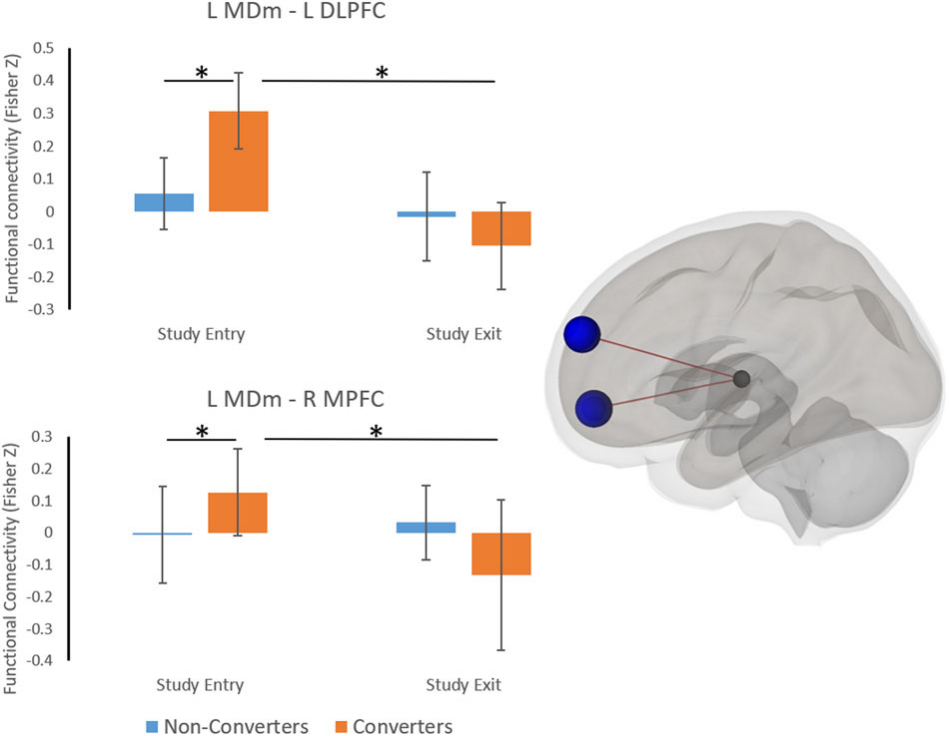
Mediodorsal thalamus functional connectivity. Functional connectivity between left mediodorsal magnacellular (MDm) and left dorsolateral prefrontal cortex (DLPFC), and right medial prefrontal cortex (MPFC) is shown at study entry and exit. At study entry, coupling between the MDm and DLPFC and MPFC was stronger in converters compared to non-converters, however this showed a significantly greater decline over the two years. Error bars indicate sample standard deviations. * P-FDR < 0.05.

At study entry (*N* = 41), significant differences in thalamo-cortical resting-state coupling were found between converters and non-converters. The mediodorsal magnacellular, lateral dorsal, anteroventral, parafascicular, centeromedian, and ventral anterior nuclei were more strongly coupled with prefrontal and cingulate associative and limbic regions and less strongly coupled with frontoparietal primary and association sensorimotor areas in converters compared to non-converters at study entry (Supplementary Table 5). Over the two years (*N* = 41), a marked decrease in coupling strength between the mediodorsal magnacellular and dorsolateral and medial prefrontal cortex was seen in converters compared to non-converters (Fig. 5).

### Validation study: Progression of thalamic local volumes in PPMI cohort

Twenty-four FOG converters and a hundred and one non-converters across a five-year follow-up were included from the PPMI cohort in this analysis. Linear mixed models evaluated the effect of group (converter vs non-converter) on local volume progression within the three thalamus clusters. No significant results were found between converters and non-converters in the two study-entry clusters on the left and right thalamus. However, for the longitudinal cluster on the left thalamus, a significant main effect of group (*p* = 0.039) was found, as well as significant interactions between years of follow-up and group (*p* = 0.001), and between its quadratic term and group (*p* = 0.048) (Supplementary Table 6). Importantly, although converters showed local deflation compared to non-converters in this cluster within the observed follow-up (see beta estimates in Supplementary Table 6), the sign for the interaction between group and years of follow-up quadratic effect was positive, signaling that local volume increased to a greater extent in converters over time (concave upward). Further, local volume in this cluster was significantly associated with lower dopamine transporter uptake in the right putamen (*p* = 0.019) and tended to be associated with lower dopamine transporter uptake in the left putamen (*p* = 0.087), but higher dopamine transporter uptake in the left caudate (*p* = 0.091).

## Discussion

This prospective longitudinal MRI study investigated whether morphological alterations in the subcortical grey matter would predict conversion to FOG. The main analysis revealed that the thalamus showed local inflations bilaterally in persons with PD who are about to develop FOG, similar to those with overt FOG. Over two years, these local inflations persisted in those participants who converted. Furthermore, a model including thalamic local volume measures could predict conversion to FOG with good accuracy. Correlation analyses further showed that local volume inflation of the thalamus was associated with larger volumes of medial thalamic sub-nuclei and better performance on tests of executive function. Finally, thalamo-cortical resting-state functional coupling was increased between various thalamic sub-nuclei and limbic and associative cortical regions prior to conversion, which declined more in converters than in non-converters over the two years. These findings provide evidence of a novel marker of conversion to FOG in PD and suggest a specific pattern of adaptive neural plasticity prior to conversion, driven through the medial thalamic nuclei.

The candidate underlying physiological mechanisms of local increases in grey matter range from neurogenesis to angiogenesis, synaptogenesis, and gliogenesis. These alterations are likely driven by various neurotransmitters and neurotrophic factors that determine structural changes in response to experience and neural challenge (for review see Zatorre et al.^37^). In persons with PD, these adaptive mechanisms are not completely lost, as grey matter increases have been reported consequent to, for example, exercise^38^ and mindfulness^39^. Post-mortem studies in PD indicate that the thalamic sub-nuclei show changes in both size and shape as compared to healthy controls^40^, with varying patterns of neuronal loss, and medial structures are typically less affected^41^. Here, we found that FOG-related local inflations were related to larger volumes in the medial thalamic sub-nuclei such as those previously reported to be more spared in PD^41^. This medial sparing possibly potentiates the adaptive role of the medial thalamus which may result in both compensatory^42^ and maladaptive^43,44^ effects seen in animal models. In our study, larger local inflations in this region were significantly associated with better performance on executive tests, in which the mediodorsal nucleus was previously shown to play a role^45^. Further, resting-state coupling was stronger between the mediodorsal nucleus and dorsolateral and medial prefrontal cortex in converters prior to conversion. These prefrontal cortices are involved with goal-directed behaviors, on which persons with PD and FOG increasingly rely to operate otherwise automatic motor commands such as gait^46,47^, suggesting that these shape changes may indeed have an adaptive nature in origin.

Through its connections with the basal ganglia, cerebellum, and cortex^48–51^, the thalamus participates in feedback and feedforward mechanisms, playing a modulatory role in integrating information across the parallel motor, cognitive and limbic circuits (for reviews see Haber and McFarland^49^ and Varela^52^). Disinhibition of the ventral posterior lateral and ventral lateral motor nuclei of the thalamus^53^ through the direct dopaminergic pathway normally leads to a release of motor responses^54^. A large prospective study showed that persons at risk of developing FOG already had reduced striatal dopamine availability^18^. Although we did not measure dopamine availability or responsiveness, significant associations were found in this cohort between local volume in the thalamus and functional impact and predictability of OFF fluctuations, which suggests failing dopaminergic control of motor symptoms. This reduction in dopamine availability or effectiveness may thus lead to a lack of disinhibition of the lateral thalamic nuclei, triggering thalamo-striatal feedback or thalamo-cortical feedforward mechanisms to augment motor output, via the medial thalamic nuclei^55^.

We also assessed the OFF-medication resting-state coupling between thalamic sub-nuclei and sub-cortical and cortical areas to investigate whether thalamic functional connectivity could possibly be driving the local shape changes. We found that medial thalamic sub-nuclei showed stronger coupling with cortical associative and limbic areas, and weaker coupling with sensorimotor areas (recall Supplementary Table 5) in converters. Furthermore, we showed that the coupling between the mediodorsal nucleus and the dorsolateral and medial prefrontal cortices of the cognitive control-network declined with time in converters. These findings corroborate and prefigure prior cross-sectional work in freezers showing increased coupling across cortico-subcortical limbic circuitry at rest^56^, and reduced coupling within the cognitive control networks at rest^56,57^ and during freezing episodes^58,59^. Our findings thereby add to the body of literature indicating that persons with PD increasingly rely on non-motor circuits^60,61^ in order to overcome reduced processing in the depleted sensorimotor circuits, and that over time, altered processing in these compensatory circuits predisposes them for gait breakdown and the onset of FOG. As a result of this increasing reliance on the limbic and associative circuitry, plastic changes may occur in the coupled medial thalamic nuclei, giving rise to the morphological differences seen here.

Besides the motor, limbic and cognitive aspects mentioned in relationship to the onset of FOG, another interesting finding was that the suprageniculate nucleus volume was inversely associated with the local thalamic shape changes both at study entry and over the two years. This nucleus plays a role in spatial localization of stimuli within the visual field^62^, which is corroborated by the strong correlations we found between these local volumes and cognitive tests loading highly on visual search such as the trail making test and the complex Figure of Rey copy task. The decline in the volume of this structure may in fact also partly explain the visuospatial deficits found pre-^16^ and post-conversion to FOG in people with PD^63,64^. One mechanism through which visual cues may mediate their effects may be through circumventing these deficits in extracting salient features from the environment, and by guiding attention to relevant information^65^.

The predictive model including local volume of the thalamus was able to predict conversion with good accuracy (bootstrap-AUC = 0.82), performing similarly to a far more extensive model based on behavioral information (bootstrap-AUC = 0.79) in the same cohort^17^. Contrary to our expectations based on cross-sectional work^29,32,33^, global volumes of subcortical structures were not found to be sensitive markers of conversion to FOG (recall Supplementary Table 1). In line with the study by Snijders and others (2011), we found smaller brainstem volumes in freezers compared to non-converters, but not between converters and non-converters at study entry. Over the two years, volumes in the brainstem, left pallidum and bilateral thalami even showed a relative increase in converters compared to non-converters, indicating that the decline in the volume of these structures reported in earlier studies^32,33^ may occur subsequent to the onset of FOG. The increase in brainstem global volume in converters was particularly unexpected, but being in accordance with the brainstem local volume changes, likely indicates a non-linear trajectory in brainstem volume over the disease course. Importantly, the local volume changes in the brainstem may reflect involvement of the mesencephalic locomotor region and the pedunculopontine nucleus (recall Fig. 2B), which along with the thalamus and pallidum are involved in sensory-guided locomotor control through the dorsal pathway^66^. Although this remains to be tested, the relative volume increase in converters may suggest a compensatory shift to external sensory-guided control of locomotion to overcome a failure of the internal motor drive.

Owing to the small sample of converters in the original study, external validation was conducted using the PPMI cohort, the largest open access database of PD neuroimaging data. However, the multi-center study did not focus on FOG and therefore lacked a validated scale to establish the presence of FOG. Further, as these participants were untreated at inclusion and medication doses were frequently adjusted, the emergence and disappearance of FOG is plausible during follow-up. We therefore took a more inclusive approach to classifying FOG, using self- or rater-reported information across all follow-up visits, despite reponses at subsequent visits. Further, as the imaging protocols (scanner hardware and software, acquisition sequences) were different from our own cohort, the local volume metrics may not be comparable. Despite these sources of variability, we showed that local volumes in one thalamus cluster showed a different progression in converters and non-converters across both cohorts, and that in de novo PD these trajectories diverge as the disease progresses. Furthermore, greater local volumes in this cluster were associated with lower dopamine transporter uptake in the putamen, but tended to be associated with higher dopamine transporter uptake in the left caudate. Putamen dopamine availability reduction likely leads to reduced locomotor automaticity, leading to a compensatory shift to more cognitive control of gait mediated through the caudate-prefrontal circuits, as long as caudate dopamine is preserved. These relationships with striatal dopamine availability lend additional support to the compensatory explanation for the thalamic structural changes.

This multimodal prospective longitudinal study addressing structural imaging markers and FOG conversion is not without its limitations. Despite the large sample of non-freezers, only 20% of participants developed FOG over two years leading to a small group of converters. Although conversion rates were low, rendering the study underpowered to detect shape changes in other FOG-related nuclei^29,67^, multi-method investigations showed a consistent pattern of results. Further, in our primary contrast, we pooled together converters and freezers in order to increase FOG specificity by looking at deficits common to these groups as well as to increase statistical power. Despite differences in disease stage, FOG classification, and imaging parameters, results from the PPMI cohort supported the patterns found, while further suggesting that the markers of conversion found would probably not be useful in the early stages of the disease. These results were expected based on the contribution of levodopa equivalent dose to the prediction model and associations between local volumes and medication fluctuations (our data), which were also replicated with the PPMI data using more direct estimates of striatal dysfunction. Longer follow-ups of the PPMI cohort may provide the opportunity to compare the cohorts at a similar disease stage. Finally, as FOG may be heterogeneous^68^, it is also possible that the results may relate to a particular phenotype of FOG. Future multi-center prospective cohort studies with comprehensive FOG characterization and longer follow-ups could overcome many of these limitations.

In summary, through this prospective longitudinal MR study, we showed that alterations in bilateral thalamus morphology predicted conversion to FOG in PD. Behavioral associations and thalamo-cortical connectivity suggested that the morphological changes were compensatory in nature, and conversion to FOG was accompanied by decompensation within these circuits over two years. These findings reveal the potential of thalamus morphology as a marker for screening individuals at risk of developing FOG and shed light on the role of the thalamus in the aetiology of FOG.

## Methods

### Participants

Fifty-seven persons (45 without FOG and 12 with FOG) with idiopathic PD (UK Parkinson’s Disease Brain Bank criteria) were recruited and prospectively followed up for two years. Participants underwent extensive behavioral testing and MRI scanning at study entry and two years later, at the Movement Analysis Laboratory of KU Leuven and the University Hospital of Leuven, Belgium. To be eligible for inclusion, participants needed to be able to walk unassisted for 10 min, be free from Deep Brain Stimulators and other MR contraindications, and without probable dementia (Mini Mental Status Examination <24) and other comorbidity. Recruitment of participants began in August 2012 and final assessments were completed in May 2016. As FOG episodes are more common when medication is withheld^69^, measurements and scans were performed in the morning while participants were “OFF” medication – at least 12 h after the last dopaminergic medication intake. In accordance with the Declaration of Helsinki, participants provided written informed consent prior to enrolment. The study was approved by the Ethics Committee Research UZ/KU Leuven (Study number: B322201215418).

### Behavioral testing

The full test battery of behavioral measures has been reported elsewhere^17^. Here, we present new neuroimaging data from this longitudinal cohort study. Global cognition was assessed with the Montreal Cognitive Assessment (MoCA)^70^, executive function with the Frontal Assessment Battery (FAB)^71^, Trail Making Test B-A time (TMTB-A), and Alternate Naming Test, and visuospatial function and working memory with the Complex Figure of Rey test. Affect was measured with the Hospital Anxiety and Depression Scale^72^. Balance performance was assessed with the Mini Balance Evaluation Test (MiniBEST)^73^ and symptom severity with the Movement Disorders Society Revision of the Unified Parkinson’s Disease Rating Scale (MDS-UPDRS)^74^. Participants were classified as having FOG by their response to the first question of the New Freezing of Gait Questionnaire (NFOG-Q) (“Did you experience Freezing episodes over the past month”), after showing them a video with different kinds of freezing episodes^75^. Importantly, none of the non-freezers showed objective freezing during the extended behavioral test battery at study entry.

### MRI acquisition

High-resolution anatomical and resting-state functional MRI (rs-fMRI) were acquired in a 3T Philips ACHIEVA MRI scanner (Best, The Netherlands). Anatomical T1-weighted scans were acquired using Turbo Field Echo sequence (duration: 383 s, TR/TE: 9.6/4.6 ms, flip angle: 8°, voxel size: 0.98 × 1.2 × 0.98 mm, number of slices: 182, FOV: 218.4 × 250 × 250 mm). Rs-fMRI scans were acquired using T2*-weighted Gradient Echo Planar Imaging sequence (duration: 435 s, TR/TE: 1700/33 ms, flip angle: 90°, voxel size: 3.59 × 3.74 × 4 mm, number of slices: 31, FOV: 230 × 124 × 230 mm). During the scan, participants had their eyes open while looking at a standard crosshair on a blank screen.

### MRI processing – shape analysis

Vertex-based analysis of fifteen subcortical structures (amygdala, nucleus accumbens, caudate, hippocampus, pallidum, putamen, thalamus – bilaterally; and brainstem) was performed using FMRIB Software Library’s (FSL, version 5.0.9)^76^ FMRIB Integrated Registration and Segmentation Tool (FIRST) (https://fsl.fmrib.ox.ac.uk/fsl/fslwiki/FIRST)^77^. This automated method has been validated in healthy and patient populations with high retest reliability over repeated measurements^78^, particularly for segmenting the striatum^79^. Deformable mesh models were fitted to the surface of each structure and local and global volumes were calculated. Local volume quantifies subregional shape variations on the surface of a nucleus – calculated as the scalar distance of an individual’s vertices to the mean sample vertices. Global volume quantifies the total volume of the nucleus – calculated as the volume within the surface mesh. A change score for each participant was calculated as Scan2 – Scan1, so positive values imply inflation in local volume or an increase in global volume over the two years.

### Data Analysis

For the main analysis, we investigated *local and global volume differences* at study entry and over the two years:

i. Using data from study entry (*N* = 57), we investigated structural alterations between non-converters (*N* = 36) and converters and freezers (*N* = 21) in order to identify FOG-specific structural markers. We then performed post-hoc analyses to test which FOG sub-group differed from non-converters. The predictive value and behavioral relationships of these markers were tested in a subsequent analysis.
ii. Using change scores over the two years (*N* = 43), we investigated structural markers showing a differential progression between non-converters (*N* = 36) and converters (*N* = 7). The relationship between these markers and behavioral change was tested in a subsequent analysis.

*Local volume alterations* were analyzed by non-parametric permutation testing^80^ using FSL’s *randomise* tool, with age, gender and daily levodopa equivalent dose as nuisance regressors. Ten thousand permutations were performed and the family-wise error (FWE) rate was corrected using Threshold Free Cluster Enhancement (two-dimensional), which has been shown to be more sensitive and robust than voxel-based or cluster-based thresholding methods^81,82^. Mean local volume in clusters showing a significant effect was extracted and post-hoc analyses were performed in SPSS v.25 (IBM Corp, NY). These constituted an analysis of covariance between non-converters, converters, and freezers with age, gender, and daily levodopa equivalent dose as covariates. Disease duration and MDS-UPDRS motor scores were considered as covariates, however, models containing these measures led to worse model fit, based on the Akaike’s Information Criteria. Pairwise comparisons were corrected with the Bonferroni method.

*Global volumes* of the fifteen subcortical structures were extracted from the segmented meshes in native space. Total intracranial volume was estimated using SPM 12 (Wellcome Trust Centre for Neuroimaging, London)^83^. The two contrasts (i and ii above) were tested with an analysis of covariance, using age, gender, daily levodopa equivalent dose and total intracranial volume as covariates. Post-hoc pairwise comparisons between non-converters, freezers and converters were also corrected with the Bonferroni method.

To investigate the *predictive performance* of the significant local volume clusters, logistic regression in the group of non-freezers (*N* = 45) at study entry (non-converters and converters) was performed. Collinearity was first assessed between all predictors including the significant clusters, age, gender and daily levodopa equivalent dose. Predictors with a variance inflation factor <4 were included in a backward logistic regression model (Wald chi-square test - P_stay_ = 0.1). The area under the receiving operator characteristics curve (AUC), Brier score^84^ and optimal performance of the model based on the Youdens’s index^85^ are reported. Validation was performed with boostrap resampling, similar to previous work^17^.

*Association of local volume measures* with clinical measures at study entry (max *N* = 45) and over two years (max *N* = 43) was investigated using Pearson’s product moment correlations in the non-converters and converters. No multiple comparison correction was performed for this exploratory analysis.

To assess *anatomical specificity* of the significant thalamic local volume findings, we investigated the association between local volumes and thalamic sub-nuclei volumes in the non-converters and converters. A recently developed method^86^ was used to segment twenty-five thalamic sub-nuclei with FreeSurfer v.6 (http://surfer.nmr.mgh.harvard.edu/)^87^. Pearson product moment correlation was performed between local volume measures and sub-nuclei volumes at study entry and between volumetric changes over two years.

To explore the *functional correlates* of the local volume changes, we also investigated Rs-fMRI functional connectivity between the thalamic sub-nuclei and sub-cortical and cortical areas at study entry and over the two years (*N* = 41 for both analyses, non-converters = 34, converters = 7). Rs-fMRI volumes were preprocessed using fMRIprep v.1.5.8^88^ (see Supplementary Methods for boilerplate and additional preprocessing details) and further denoising and ROI-to-ROI analyses were performed using the CONN toolbox v.19b^89^. Seed-to-target coupling was compared between non-converters and converters at study entry and over two years using an analysis of covariance with age, sex, and daily levodopa equivalent dose included as covariates. Multiple comparisons for each seed region were corrected with the (Benjamini and Hochberg) false discovery rate procedure.

Finally, to validate these findings, the progression of local volumes in the PPMI dataset were investigated. Clinical and structural MRI data were obtained from the PPMI database, downloaded on the 29th of June 2018. For up-to-date information on the PPMI study and data, visit www.ppmi-info.org. To be eligible for inclusion, participants had to have a diagnosis of PD for 2 years or less and be unmedicated. Participants from this de-novo PD cohort for whom 3T MR high resolution (1mm^3^) anatomical scans were available, and who did not present with FOG at diagnosis were included in the present analysis. Conversion classification was based on the MDS-UPDRS scores, with either consistent subjective reports of FOG on item 2.13 at multiple (>1) follow-ups or an objective rating of FOG on item 3.11(OFF-medication) at any follow-up visit. Shape analysis was performed similarly as described above for the MRI data of the present study for 388 scans from 125 participants, 24 of whom were classified as converters. Median follow-up was 4 years, with anatomic and dopamine transporter scans having been acquired at baseline, one-year, two-year, and four-year follow-ups. Thalamus clusters that were significant in our own cohort (left and right thalamus at study entry and left thalamus over the two years) were used as masks to extract the local volumes in the PPMI cohort.

Linear mixed models were then used to investigate differences in the cluster local volumes between PPMI converters and non-converters over time. Specifically, the models were run separately for the three thalamus clusters’ local volumes as dependent variables with years of follow-up, its quadratic term and group, and their interactions as independent variables. Age, sex, and left and right caudate and putamen dopamine transporter uptake were included as covariates. A random intercept was estimated for participants within each center, with a variance component. Fit of the models was visually assessed. If we would find significant group effects or significant interaction effects between group and years of follow-up or its quadratic term, these would indicate external validity of local volume within these clusters as predictors of conversion to FOG.

### Reporting summary

Further information on research design is available in the Nature Research Reporting Summary linked to this article.

## Supplementary information

Supplementary Tables and Methods

Nature Reporting Summary

## Data Availability

The data used in this manuscript can be provided upon reasonable request from the author and in agreement with the KU Leuven.

## Code availability

All neuroimaging analyses performed in this manuscript were performed with freely available neuroimaging tools namely FSL, FreeSurfer, fMRIPrep, ANTs, CONN toolbox, and SPM. CONN toolbox was implemented in a Matlab environment (version 16b, The MathWorks, Natick, MA, USA) and statistical analyses for internal and external validation were performed in SAS version 9.4 for Windows (SAS Institute Inc., Cary, NC, USA). Code for specific processes can be obtained from the author upon reasonable request.
