# Supplementary material for: Thalamic morphology predicts the onset of freezing of gait in Parkinson’s disease
Source: NPJ Parkinsons Dis. 2021 Mar 2;7:20. doi: 10.1038/s41531-021-00163-0 (PMC7925565; doi:10.1038/s41531-021-00163-0)
Supplement: Supplementary file 1 — Supplementary Tables and Methods [file 41531_2021_163_MOESM1_ESM.pdf]

Supplementary Table 1. Local volume differences at study entry and over two years. Clusters where local volume differences were significant, corrected for age, gender and daily levodopa equivalent dose, thresholded at $P_{FWE} < .05$ for analyses at study entry and at $P_{FWE} < .1$ for analyses over two years. A – between non-converters and freezers and converters at study entry, B – between non-converters and converters over two years.

**Region**	***Location***	**Size (voxels)**	**P (min)**	**Center Coordinates (MNI)**		
				**X**	**Y**	**Z**
A. At study entry						
Non-converters < freezers + converters						
Left Thalamus	*Medial and lateral*	1126	.006	-11	-22	7
Right Thalamus	*Medial and lateral*	917	.012	13	-22	7
B. Change over two years						
Non-converters < converters						
Brainstem	*Posterior Midbrain*	433	.045	-1	-39	-16
Left Thalamus	*Lateral*	150	.060	-15	-11	5
Left Amygdala	*Medial Inferior*	66	.083	-18	-7	20

Supplementary Table 2. Bootstrap resampling of predictive model selection and evaluation. One thousand resamples with replacement were made from the original dataset and in each resample, backward selection was used to fit a logistic regression model (P-stay = .1). The performance of the fitted model was evaluated in the original dataset and estimates were averaged and reported below.

**Model performance**	
AUC	0.82
Youden's Index (YI) cutoff	0.2
Accuracy at YI	78.6%
Sensitivity at YI	76%
Specificity at YI	79%
Brier Score	0.12

**Parameter Selection Frequency (% models)**	
Age	28.7%
Gender	66.7%
Levodopa Equivalent Dose	96.3%
Left thalamus local volume	56.8%
Right thalamus local volume	36.5%

Supplementary Table 3. Bivariate Pearson correlation coefficient between clinical measures and local volumes in the significant clusters at study entry. Only non-freezers at study entry (non-converters and converters) were included in this analysis. * - P < .05, ** - P < .01.

Variable	Left Thalamus	Right Thalamus
Disease Severity		
Daily Levodopa Equivalent Dose	-.264	-.173
MDS-UPDRS Part I Non-Motor ADL	.023	.05
MDS-UPDRS Part II Motor ADL	-.215	-.111
MDS-UPDRS Part III Motor Exam	.050	.232
MDS-UPDRS Part IV Motor Complications	.430**	.436**
Cognition		
Montreal Cognitive Assessment total score	.246	.205
Trail Making Test time (Part B minus Part A)	-.342*	-.380*
Alternate Naming Test time	-.426**	-.320*
Frontal Assessment Battery total score	.277	.264
Figure of Rey Copy time	-.329*	-.404**
Figure of Rey Recall score	.319*	.262
Affect & Balance		
Hospital Anxiety and Depression - Anxiety score	.017	-.015
Hospital Anxiety and Depression - Depression score	-.125	-.008
MiniBEST total score	.210	.255

Supplementary Table 4. Bivariate Pearson's correlation coefficient between right and left thalamus sub-nuclei volumes and local volumes in the two clusters at study entry (first four columns) and with change scores in sub-nuclei volumes and the left thalamus cluster over the two years (last two columns). Only non-freezers at study entry were included for the analysis at study entry (N = 45) and over two years (N = 43). * - P < .05, ** - P < .01

Sub-nuclei	Left thalamus local volume		Right thalamus local volume		Left thalamus local volume change	
	Left	Right	Left	Right	Left	Right
Gross volume	.189	.205	.190	.237	.185	.242
Anteroventral	.225	.295*	.308*	.510**	.146	.057
Central Medial	.435**	.494**	.468**	.593**	.135	.156
Central Lateral	.113	.294*	.099	.471**	.175	.123
Centromedian	-.063	.012	-.078	.014	.145	.180
Laterodorsal	.176	.306*	.212	.473**	.282	.181
Lateral geniculate	.067	.163	.036	.038	.202	.127
Lateral posterior	-.086	.079	-.100	.220	.202	.255
Limitans (suprageniculate)	-.266	-.292	-.202	-.299*	-.213	-.434**
Mediodorsal medial parvocellular	.158	.122	.214	.144	-.037	-.053
Mediodorsal medial magnocellular	.445**	.412**	.463**	.511**	-.031	.022
Medial Geniculate	.208	.166	.166	.053	.102	.040
Reuniens (medial ventral)	.446**	.503**	.547**	.622**	.000	.154
Paracentral	.398**	.278	.363*	.346*	.314*	.034
Parafascicular	-.226	.036	-.182	.059	.152	.185
Paratenial	-.123	-.048	-.007	.030	-.008	-.135
Pulvinar Anterior	.300*	.392**	.276	.391**	.158	.015
Pulvinar Inferior	.033	.084	-.060	-.017	.061	.185
Pulvinar Lateral	.086	-.045	.094	-.167	-.002	-.028
Pulvinar Medial	.180	.238	.110	.199	.164	.216
Ventral anterior	.129	.036	.134	.147	-.041	.030
Ventral anterior magnocellular	.168	.245	.151	.344*	.069	.002
Ventral lateral anterior	.140	.095	.157	.160	-.032	.249
Ventral lateral posterior	.112	.079	.149	.133	.169	.175
Ventromedial	-.108	-.095	-.092	-.139	-.013	.207
Ventral posterolateral	-.127	-.066	-.082	-.099	.146	.144

Supplementary Table 5. Differences in thalamo-cortical resting state functional connectivity between non-converters and converters at study entry and over two years. Effect size (beta value), T-statistic (two-tailed), uncorrected and false discovery rate corrected p-values are shown. Beta values and T-statistics are always in the direction of converters. Age, gender and daily levodopa equivalent dose were entered as covariates.

Seed		Target		Beta	T(36)	P-unc	P-FDR
Study Entry: Non-Converters < Converters							
Right	Parafascicular	Left	Cerebellum	.19	4.51	<.001	.025
Right	Parafascicular	Right	Posterior Cingulate Cortex	.16	3.85	<.001	.038
Left	Mediodorsal magnacellular	Left	Dorsolateral Prefrontal Cortex	.26	5.11	<.001	.004
Left	Mediodorsal magnacellular	Left	Dorsolateral Prefrontal Cortex	.31	4.12	<.001	.040
Left	Mediodorsal magnacellular	Left	Paralimbic Cortex	.21	3.93	<.001	.046
Right	Anteroventral	Left	Posterior Cingulate Cortex	.18	3.56	.001	.021
Right	Anteroventral	Left	Middle Cingulate Cortex	.16	3.35	.002	.026
Right	Anteroventral	Left	Posterior Cingulate Cortex	.19	3.3	.002	.029
Right	Anteroventral	Left	Middle Cingulate Cortex	.18	3.15	.003	.035
Right	Anteroventral	Left	Posterior Cingulate Cortex	.15	3.13	.004	.036
Right	Anteroventral	Left	Putamen	.19	3.04	.004	.040
Right	Anteroventral	Left	Posterior Cingulate Cortex	.14	2.93	.006	.048
Right	Anteroventral	Right	Thalamus	.28	4.23	<.001	.009
Right	Anteroventral	Right	Ventral Diencephalon	.17	3.87	<.001	.014
Right	Anteroventral	Right	Paralimbic Cortex	.17	3.56	.001	.021
Right	Anteroventral	Right	Posterior Cingulate Cortex	.2	3.2	.003	.034
Right	Anteroventral	Right	Posterior Cingulate Cortex	.17	3.15	.003	.035
Right	Anteroventral	Right	Middle Cingulate Cortex	.19	3.1	.004	.038
Right	Anteroventral	Right	Dorsolateral Prefrontal Cortex	.2	3.05	.004	.040
Right	Anteroventral	Right	Middle Cingulate Cortex	.14	2.96	.005	.047
Right	Anteroventral	Right	Anterior Cingulate Cortex	.17	2.92	.006	.048
Study Entry: Non-Converters > Converters							
Right	Ventral Anterior	Right	Primary Somatosensory Cortex	-.25	-4.57	<.001	.021
Left	Parafascicular	Left	Inferior Frontal Sulcus	-.13	-4.05	<.001	.049
Right	Parafascicular	Left	Insula Granular Cortex	-.2	-3.82	.001	.038
Right	Parafascicular	Left	Retroinsular Auditory Cortex	-.17	-3.71	.001	.039
Right	Parafascicular	Left	Posterior Opercular Cortex	-.17	-3.61	.001	.039
Left	Parafascicular	Right	Auditory Association Cortex	-.18	-4.38	<.001	.036
Right	Parafascicular	Right	Medial Superior Temporal Cortex	-.16	-4.05	<.001	.038
Right	Parafascicular	Right	Dorsal Stream Visual Cortex	-.15	-3.91	<.001	.038
Right	Parafascicular	Right	Middle Temporal Cortex	-.15	-3.66	.001	.039
Right	Parafascicular	Right	Posterior Opercular Cortex	-.15	-3.62	.001	.039
Left	Lateral Geniculate	Right	Middle Temporal Gyrus	-.16	-4.34	<.001	.026
Left	Lateral Geniculate	Right	Orbitofrontal Cortex	-.15	-4.26	<.001	.026
Right	Centeromedian	Left	Orbitofrontal Cortex	-.18	-4.88	<.001	.008
Right	Anteroventral	Left	Primary Somatosensory Cortex	-.28	-4.91	<.001	.008

Right	Anteroventral	Left	Dorsal Stream Visual Cortex	-.24	-4.47	<.001	.009
Right	Anteroventral	Left	Superior Parietal Cortex	-.24	-4.34	<.001	.009
Right	Anteroventral	Left	Superior Parietal Cortex	-.19	-4.22	<.001	.009
Right	Anteroventral	Left	Posterior Temporal Cortex	-.23	-4.16	<.001	.009
Right	Anteroventral	Left	Primary Somatosensory Cortex	-.23	-3.73	.001	.016
Right	Anteroventral	Left	Primary Somatosensory Cortex	-.25	-3.5	.001	.023
Right	Anteroventral	Left	Inferior Parietal Cortex	-.22	-3.49	.001	.023
Right	Anteroventral	Left	Superior Parietal Cortex	-.16	-3.45	.001	.023
Right	Anteroventral	Left	Premotor Cortex	-.17	-3.41	.002	.025
Right	Anteroventral	Left	Medial Superior Temporal Cortex	-.2	-3.23	.003	.033
Right	Anteroventral	Left	Superior Parietal Cortex	-.17	-3.19	.003	.035
Right	Anteroventral	Left	Superior Parietal Cortex	-.16	-3.08	.004	.039
Right	Anteroventral	Left	Primary Somatosensory Cortex	-.17	-2.98	.005	.046
Right	Anteroventral	Left	Posterior Opercular Cortex	-.18	-2.95	.006	.048
Right	Anteroventral	Left	Inferior Parietal Cortex	-.17	-2.93	.006	.048
Right	Anteroventral	Right	Primary Somatosensory Cortex	-.26	-4.45	<.001	.009
Right	Anteroventral	Right	Superior Parietal Cortex	-.19	-4.2	<.001	.009
Right	Anteroventral	Right	Inferior Parietal Cortex	-.23	-3.99	<.001	.013
Right	Anteroventral	Right	Premotor Cortex	-.25	-3.94	<.001	.013
Right	Anteroventral	Right	Superior Parietal Cortex	-.2	-3.93	<.001	.013
Right	Anteroventral	Right	Primary Somatosensory Cortex	-.25	-3.77	.001	.016
Right	Anteroventral	Right	Superior Parietal Cortex	-.2	-3.73	.001	.016
Right	Anteroventral	Right	Medial Superior Temporal Cortex	-.23	-3.71	.001	.016
Right	Anteroventral	Right	Primary Somatosensory Cortex	-.25	-3.59	.001	.021
Right	Anteroventral	Right	Primary Motor Cortex	-.25	-3.46	.001	.023
Right	Anteroventral	Right	Lateral Occipital Cortex	-.18	-3.4	.002	.025
Right	Anteroventral	Right	Temporo-Parieto-Occipital Junction	-.18	-3.37	.002	.026
Right	Anteroventral	Right	Superior Parietal Cortex	-.13	-3.27	.002	.031
Right	Anteroventral	Right	Primary Somatosensory Cortex	-.21	-3.15	.003	.035
Right	Anteroventral	Right	Superior Parietal Cortex	-.18	-3.07	.004	.039
Change over two years: Non-Converters < Converters							
Left	Suprageniculate	Right	Posterior Cingulate Cortex	.23	4.41	<.001	.033
Left	Pulvinar Inferior	Right	Premotor Cortex	.22	4.7	<.001	.014
Right	Parafascicular	Right	Posterior Insular Cortex	.28	4.9	<.001	.008
Left	Parafascicular	Right	Primary Somatosensory Cortex	.24	4.28	<.001	.050
Left	Lateral Geniculate	Right	Middle Temporal Gyrus	.22	4.86	<.001	.009
Change over two years: Non-Converters > Converters							
Left	Mediodorsal magnacellular	Left	Dorsolateral Prefrontal Cortex	-.34	-4.21	<.001	.030
Left	Mediodorsal magnacellular	Right	Medial Prefrontal Cortex	-.34	-4.28	<.001	.030
Left	Lateral Posterior	Left	Superior Parietal Cortex	-.21	-4.5	<.001	.026

Supplementary Table 6. PPMI validation cohort. Linear mixed model estimates, 95% confidence intervals (CI) and probability values for the left thalamus change cluster for all terms included in the model.

**Effect**	**Estimate**	**95% CI Lower**	**95% CI Upper**	**P**
Intercept	1.141	.947	1.336	<.0001
Age	-.016	-.019	-.014	**<.0001**
Sex (male vs female)	-.125	-.176	-.073	**<.0001**
Right Caudate DAT DVR	.004	-.031	.039	.818
Left Caudate DAT DVR	.030	-.005	.065	.091
Right Putamen DAT DVR	-.048	-.088	-.008	**.019**
Left Putamen DAT DVR	-.043	-.092	.006	.087
Years of follow-up	.008	.001	.015	**.024**
Years of follow-up^2	-.001	-.004	.003	.658
Group (converters vs non-converters)	-.079	-.155	-.004	**.039**
Years of follow-up * Group (converters vs non-converters)	-.018	-.029	-.007	**.001**
Years of follow-up^2 * Group (converters vs non-converters)	.007	.0001	.014	**.048**

Supplementary Methods – Resting State Analysis

Results included in this manuscript come from preprocessing performed using fMRIPrep 1.5.8 (Esteban, Markiewicz, et al. (2018); Esteban, Blair, et al. (2018); RRID:SCR_016216), which is based on Nipype 1.4.1 (Gorgolewski et al. (2011); Gorgolewski et al. (2018); RRID:SCR_002502).

Anatomical data preprocessing

The T1-weighted (T1w) image was corrected for intensity non-uniformity (INU) with N4BiasFieldCorrection (Tustison et al. 2010), distributed with ANTs 2.2.0 (Avants et al. 2008, RRID:SCR_004757), and used as T1w-reference throughout the workflow. The T1w-reference was then skull-stripped with a Nipype implementation of the antsBrainExtraction.sh workflow (from ANTs), using OASIS30ANTs as target template. Brain tissue segmentation of cerebrospinal fluid (CSF), white-matter (WM) and gray-matter (GM) was performed on the brain-extracted T1w using fast (FSL 5.0.9, RRID:SCR_002823, Zhang, Brady, and Smith 2001). Brain surfaces were reconstructed using recon-all (FreeSurfer 6.0.1, RRID:SCR_001847, Dale, Fischl, and Sereno 1999), and the brain mask estimated previously was refined with a custom variation of the method to reconcile ANTs-derived and FreeSurfer-derived segmentations of the cortical gray-matter of Mindboggle (RRID:SCR_002438, Klein et al. 2017). Volume-based spatial normalization to two standard spaces (MNI152NLin2009cAsym, MNI152NLin6Asym) was performed through nonlinear registration with antsRegistration (ANTs 2.2.0), using brain-extracted versions of both T1w reference and the T1w template. The following templates were selected for spatial normalization: ICBM 152 Nonlinear Asymmetrical template version 2009c [Fonov et al. (2009), RRID:SCR_008796; TemplateFlow ID: MNI152NLin2009cAsym], FSL's MNI ICBM 152 non-linear 6th Generation Asymmetric Average Brain Stereotaxic Registration Model [Evans et al. (2012), RRID:SCR_002823; TemplateFlow ID: MNI152NLin6Asym].

Functional data preprocessing

For each of the 1 BOLD runs found per subject (across all tasks and sessions), the following preprocessing was performed. First, a reference volume and its skull-stripped version were generated using a custom methodology of fMRIPrep. A B0-nonuniformity map (or fieldmap) was estimated based on a phase-difference map calculated with a dual-echo GRE (gradient-recall echo) sequence, processed with a custom workflow of

SDCFlows inspired by the epidewarp.fsl script and further improvements in HCP Pipelines (Glasser et al. 2013). The fieldmap was then co-registered to the target EPI (echo-planar imaging) reference run and converted to a displacements field map (amenable to registration tools such as ANTs) with FSL's fugue and other SDCflows tools. Based on the estimated susceptibility distortion, a corrected EPI (echo-planar imaging) reference was calculated for a more accurate co-registration with the anatomical reference. The BOLD reference was then co-registered to the T1w reference using bbregister (FreeSurfer) which implements boundary-based registration (Greve and Fischl 2009). Co-registration was configured with six degrees of freedom. Head-motion parameters with respect to the BOLD reference (transformation matrices, and six corresponding rotation and translation parameters) are estimated before any spatiotemporal filtering using mcflirt (FSL 5.0.9, Jenkinson et al. 2002). BOLD runs were slice-time corrected using 3dTshift from AFNI 20160207 (Cox and Hyde 1997, RRID:SCR_005927). The BOLD time-series, were resampled to surfaces on the following spaces: fsaverage5. The BOLD time-series (including slice-timing correction when applied) were resampled onto their original, native space by applying a single, composite transform to correct for head-motion and susceptibility distortions. These resampled BOLD time-series will be referred to as preprocessed BOLD in original space, or just preprocessed BOLD. The BOLD time-series were resampled into several standard spaces, correspondingly generating the following spatially-normalized, preprocessed BOLD runs: MNI152NLin2009cAsym, MNI152NLin6Asym. First, a reference volume and its skull-stripped version were generated using a custom methodology of fMRIPrep. Automatic removal of motion artifacts using independent component analysis (ICA-AROMA, Pruim et al. 2015) was performed on the preprocessed BOLD on MNI space time-series after removal of non-steady state volumes and spatial smoothing with an isotropic, Gaussian kernel of 6mm FWHM (full-width half-maximum). Corresponding "non-aggresively" denoised runs were produced after such smoothing. Additionally, the "aggressive" noise-regressors were collected and placed in the corresponding confounds file. Several confounding time-series were calculated based on the preprocessed BOLD: framewise displacement (FD), DVARS and three region-wise global signals. FD and DVARS are calculated for each functional run, both using their implementations in Nipype (following the definitions by Power et al. 2014). The three global signals are extracted within the CSF, the WM, and the whole-brain masks. Additionally, a set of physiological regressors were extracted to allow for component-based noise correction (CompCor, Behzadi et al. 2007). Principal components are estimated after

high-pass filtering the preprocessed BOLD time-series (using a discrete cosine filter with 128s cut-off) for the two CompCor variants: temporal (tCompCor) and anatomical (aCompCor). tCompCor components are then calculated from the top 5% variable voxels within a mask covering the subcortical regions. This subcortical mask is obtained by heavily eroding the brain mask, which ensures it does not include cortical GM regions. For aCompCor, components are calculated within the intersection of the aforementioned mask and the union of CSF and WM masks calculated in T1w space, after their projection to the native space of each functional run (using the inverse BOLD-to-T1w transformation). Components are also calculated separately within the WM and CSF masks. For each CompCor decomposition, the k components with the largest singular values are retained, such that the retained components' time series are sufficient to explain 50 percent of variance across the nuisance mask (CSF, WM, combined, or temporal). The remaining components are dropped from consideration. The head-motion estimates calculated in the correction step were also placed within the corresponding confounds file. The confound time series derived from head motion estimates and global signals were expanded with the inclusion of temporal derivatives and quadratic terms for each (Satterthwaite et al. 2013). Frames that exceeded a threshold of 0.5 mm FD or 1.5 standardised DVARS were annotated as motion outliers. All resamplings can be performed with a single interpolation step by composing all the pertinent transformations (i.e. head-motion transform matrices, susceptibility distortion correction when available, and co-registrations to anatomical and output spaces). Gridded (volumetric) resamplings were performed using antsApplyTransforms (ANTs), configured with Lanczos interpolation to minimize the smoothing effects of other kernels (Lanczos 1964). Non-gridded (surface) resamplings were performed using mri_vol2surf (FreeSurfer).

Many internal operations of fMRIPrep use Nilearn 0.6.1 (Abraham et al. 2014, RRID:SCR_001362), mostly within the functional processing workflow. For more details of the pipeline, see the section corresponding to workflows in fMRIPrep's documentation.

Copyright Waiver

The above boilerplate text was automatically generated by fMRIPrep with the express intention that users should copy and paste this text into their manuscripts unchanged. It is released under the CC0 license.

Additional Preprocessing

Volumes were scrubbed if the frame-wise displacement exceeded 0.5 mm or the temporal derivative of the root mean square variance of the blood oxygen-level dependent signal was an outlier– that is, if it exceeded the 75th percentile plus three times the interquartile range (Power *et al.*, 2012). Complete scans were excluded if maximum frame-wise displacement exceeded 5 mm, or if less than four minutes of data remained after scrubbing. Two non-converters and two converters were excluded due to high-motion censoring. Based on recent evaluations of motion correction strategies (Parkes *et al.*, 2018; Satterthwaite *et al.*, 2019), motion correction and denoising involved ICA-AROMA (Pruim *et al.*, 2015), implemented with FSL's reg_filt. This was followed by compound regression of motion realignment parameters, their first order temporal derivatives and their quadratic and cubic effects (36 parameters), average white matter, CSF and grey matter (equivalent to global signal) time series and scrubbing regressors implemented in CONN toolbox. Time series also underwent linear de-trending to correct drift, and high-pass temporal filtering at 0.008 Hz post-regression. Thalamic sub-nuclei segmentations were used as participant-specific seed regions. Parcellations of the cortex based on the Human Connectome Project multimodal atlas (HCP-MMP1.0) (Glasser *et al.*, 2016) which were constructed using FreeSurfer and a custom script (Neurolab, 2018), were used as participant-specific target regions. Seed-to-target coupling was analyzed between non-converters and converters at study entry and over two years using an analysis of covariance with age, sex and daily levodopa equivalent dose included as covariates. Multiple comparisons for each seed region were corrected with the false discovery rate procedure (Benjamini and Hochberg, 1995).

Supplementary References

Abraham, Alexandre, Fabian Pedregosa, Michael Eickenberg, Philippe Gervais, Andreas Mueller, Jean Kossaifi, Alexandre Gramfort, Bertrand Thirion, and Gael Varoquaux. 2014. "Machine Learning for Neuroimaging with Scikit-Learn." Frontiers in Neuroinformatics 8. https://doi.org/10.3389/fninf.2014.00014.

Avants, B.B., C.L. Epstein, M. Grossman, and J.C. Gee. 2008. "Symmetric Diffeomorphic Image Registration with Cross-Correlation: Evaluating Automated Labeling of Elderly and Neurodegenerative Brain." Medical Image Analysis 12 (1): 26–41. https://doi.org/10.1016/j.media.2007.06.004.

Behzadi, Yashar, Khaled Restom, Joy Liau, and Thomas T. Liu. 2007. "A Component Based Noise Correction Method (CompCor) for BOLD and Perfusion Based fMRI." NeuroImage 37 (1): 90–101. https://doi.org/10.1016/j.neuroimage.2007.04.042.

Cox, Robert W., and James S. Hyde. 1997. "Software Tools for Analysis and Visualization of fMRI Data." NMR in Biomedicine 10 (4-5): 171–78. https://doi.org/10.1002/(SICI)1099-1492(199706/08)10:4/5<171::AID-NBM453>3.0.CO;2-L.

Dale, Anders M., Bruce Fischl, and Martin I. Sereno. 1999. "Cortical Surface-Based Analysis: I. Segmentation and Surface Reconstruction." NeuroImage 9 (2): 179–94. https://doi.org/10.1006/nimg.1998.0395.

Esteban, Oscar, Ross Blair, Christopher J. Markiewicz, Shoshana L. Berleant, Craig Moodie, Feilong Ma, Ayse Ilkay Isik, et al. 2018. "FMRIPrep." Software. Zenodo. https://doi.org/10.5281/zenodo.852659.

Esteban, Oscar, Christopher Markiewicz, Ross W Blair, Craig Moodie, Ayse Ilkay Isik, Asier Erramuzpe Aliaga, James Kent, et al. 2018. "fMRIPrep: A Robust Preprocessing Pipeline for Functional MRI." Nature Methods. https://doi.org/10.1038/s41592-018-0235-4.

Evans, AC, AL Janke, DL Collins, and S Baillet. 2012. "Brain Templates and Atlases." NeuroImage 62 (2): 911–22. https://doi.org/10.1016/j.neuroimage.2012.01.024.

Fonov, VS, AC Evans, RC McKinstry, CR Almli, and DL Collins. 2009. "Unbiased Nonlinear Average Age-Appropriate Brain Templates from Birth to Adulthood." NeuroImage 47, Supplement 1: S102. https://doi.org/10.1016/S1053-8119(09)70884-5.

Glasser, Matthew F., Stamatios N. Sotiropoulos, J. Anthony Wilson, Timothy S. Coalson, Bruce Fischl, Jesper L. Andersson, Junqian Xu, et al. 2013. "The Minimal Preprocessing Pipelines for the Human Connectome Project." NeuroImage, Mapping the connectome, 80: 105–24. https://doi.org/10.1016/j.neuroimage.2013.04.127.

Gorgolewski, K., C. D. Burns, C. Madison, D. Clark, Y. O. Halchenko, M. L. Waskom, and S. Ghosh. 2011. "Nipype: A Flexible, Lightweight and Extensible Neuroimaging Data Processing Framework in Python." Frontiers in Neuroinformatics 5: 13. https://doi.org/10.3389/fninf.2011.00013.

Gorgolewski, Krzysztof J., Oscar Esteban, Christopher J. Markiewicz, Erik Ziegler, David Gage Ellis, Michael Philipp Notter, Dorota Jarecka, et al. 2018. "Nipype." Software. Zenodo. https://doi.org/10.5281/zenodo.596855.

Greve, Douglas N, and Bruce Fischl. 2009. "Accurate and Robust Brain Image Alignment Using Boundary-Based Registration." NeuroImage 48 (1): 63–72. https://doi.org/10.1016/j.neuroimage.2009.06.060.

Jenkinson, Mark, Peter Bannister, Michael Brady, and Stephen Smith. 2002. "Improved Optimization for the Robust and Accurate Linear Registration and Motion Correction of Brain Images." NeuroImage 17 (2): 825–41. https://doi.org/10.1006/nimg.2002.1132.

Klein, Arno, Satrajit S. Ghosh, Forrest S. Bao, Joachim Giard, Yrjö Häme, Eliezer Stavsky, Noah Lee, et al. 2017. "Mindboggling Morphometry of Human Brains." PLOS Computational Biology 13 (2): e1005350. https://doi.org/10.1371/journal.pcbi.1005350.

Lanczos, C. 1964. "Evaluation of Noisy Data." Journal of the Society for Industrial and Applied Mathematics Series B Numerical Analysis 1 (1): 76–85. https://doi.org/10.1137/0701007.

Power, Jonathan D., Anish Mitra, Timothy O. Laumann, Abraham Z. Snyder, Bradley L. Schlaggar, and Steven E. Petersen. 2014. "Methods to Detect, Characterize, and Remove Motion Artifact in Resting State fMRI." NeuroImage 84 (Supplement C): 320–41. https://doi.org/10.1016/j.neuroimage.2013.08.048.

Pruim, Raimon H. R., Maarten Mennes, Daan van Rooij, Alberto Llera, Jan K. Buitelaar, and Christian F. Beckmann. 2015. "ICA-AROMA: A Robust ICA-Based Strategy for Removing Motion Artifacts from fMRI Data." NeuroImage 112 (Supplement C): 267–77. https://doi.org/10.1016/j.neuroimage.2015.02.064.

Satterthwaite, Theodore D., Mark A. Elliott, Raphael T. Gerraty, Kosha Ruparel, James Loughead, Monica E. Calkins, Simon B. Eickhoff, et al. 2013. “An improved framework for confound regression and filtering for control of motion artifact in the preprocessing of resting-state functional connectivity data.” NeuroImage 64 (1): 240–56. https://doi.org/10.1016/j.neuroimage.2012.08.052.

Tustison, N. J., B. B. Avants, P. A. Cook, Y. Zheng, A. Egan, P. A. Yushkevich, and J. C. Gee. 2010. “N4ITK: Improved N3 Bias Correction.” IEEE Transactions on Medical Imaging 29 (6): 1310–20. https://doi.org/10.1109/TMI.2010.2046908.

Zhang, Y., M. Brady, and S. Smith. 2001. “Segmentation of Brain MR Images Through a Hidden Markov Random Field Model and the Expectation-Maximization Algorithm.” IEEE Transactions on Medical Imaging 20 (1): 45–57. https://doi.org/10.1109/42.906424.

Benjamini Y, Hochberg Y. Controlling the False Discovery Rate: A Practical and Powerful Approach to Multiple Testing. J R Stat Soc Ser B 1995; 57: 289–300.

Glasser MF, Coalson TS, Robinson EC, Hacker CD, Harwell J, Yacoub E, et al. A multi-modal parcellation of human cerebral cortex. Nature 2016; 536: 171–178.

Neurolab C. HCP-MMP1.0 volumetric (NIfTI) masks in native structural space [Internet]. 2018Available from: https://doi.org/10.6084/m9.figshare.4249400.v5

Parkes L, Fulcher B, Yücel M, Fornito A. An evaluation of the efficacy, reliability, and sensitivity of motion correction strategies for resting-state functional MRI. Neuroimage 2018; 171: 415–436.

Power JD, Barnes KA, Snyder AZ, Schlaggar BL, Petersen SE. Spurious but systematic correlations in functional connectivity MRI networks arise from subject motion. Neuroimage 2012; 59: 2142–2154.

Pruim RHR, Mennes M, van Rooij D, Llera A, Buitelaar JK, Beckmann CF. ICA-AROMA: A robust ICA-based strategy for removing motion artifacts from fMRI data. Neuroimage 2015; 112: 267–277.

Satterthwaite TD, Ciric R, Roalf DR, Davatzikos C, Bassett DS, Wolf DH. Motion artifact in studies of functional connectivity: Characteristics and mitigation strategies. Hum Brain Mapp 2019
